# Cardiac resynchronisation therapy in dextrocardia with situs Inversus totalis: a case report of strategies and outcomes in bipolar lead-based devices

**DOI:** 10.1093/ehjcr/ytag167

**Published:** 2026-03-10

**Authors:** Sujoy Khasnavis, Samer Saouma, Michael Grushko, Jay Gross

**Affiliations:** Internal Medicine, Jacobi North Central Bronx Hospital, 3424 Kossuth Avenue, Bronx, NY 10467, USA; Cardiology, Montefiore Medical Center, 111 East 210th St, Bronx, NY 10467, USA; Internal Medicine, Jacobi North Central Bronx Hospital, 3424 Kossuth Avenue, Bronx, NY 10467, USA; Cardiology, Montefiore Medical Center, 111 East 210th St, Bronx, NY 10467, USA

**Keywords:** Dextrocardia, Multipolar catheter, Right-sided CSC system, Bipolar CS lead, Situs inversus totalis, Cardiac resynchronisation therapy pacemaker, Case report

## Abstract

**Background:**

Cardiac resynchronisation therapy pacemaker (CRT-P) placement and coronary sinus cannulation (CSC) are notably challenging in dextrocardia (DXC).

**Case summary:**

A 56-year-old female with a diagnosis of DXC, sick sinus syndrome (SSS), paroxysmal atrial fibrillation (pAF), and heart failure reduced ejection fraction (HFrEF) presented for symptoms with exertion. Electrocardiogram (ECG) showed prolonged QRS necessitating CRT-P and CSC with right sided multipolar catheter and a bipolar lead. Postoperatively, there was improvement in QRS, EF, and symptoms.

**Discussion:**

CRT-P and CSC techniques in levocardia are well established. DXC requires unconventional CRT-P strategies. A right-sided multipolar catheter is valuable for CSC in DXC. This DXC case utilized a right-sided electrophysiology catheter and a bipolar coronary sinus (CS) lead. The use of these devices ensured that if the prior right ventricular (RV) ICD lead failed, a standard high S1 lead would be compatible with pacemaker headers.

**Take Home Message:**

A right-sided CSC system is useful for implanting CS leads with a left-sided device in DXC. While it comes at the expense of losing different pacing vectors, a bipolar CS lead is essential if there is a risk that the pre-existing bipolar RV lead will fail.

Learning points

- In dextrocardia with situs inversus totalis, the presence of a left-sided cardiac device makes it practical to utilize a right-sided coronary sinus cannulation system for cardiac resynchronisation therapy pacemaker placement.
- The use of bipolar coronary sinus leads is advantageous if there is concern for right ventricular lead failure and compatibility issues.

## Introduction

Dextrocardia (DXC) with situs inversus totalis (SIT) is a phenomenon known for its reversal of internal organ placement in the body.^1–4^ As such, the locations of major vessels, including the superior vena cava, are typically reversed in location with placement on the left of the reversed heart.^4^ Moreover, conduction system disease has also been reported to coexist in cases of dextrocardia.^4^ Although therapeutic options for the treatment of arrhythmias in levocardia are abundant and viable, these options are technically more challenging in DXC with SIT.^4^ Among these therapies is cardiac resynchronisation therapy pacemaker (CRT-P), which has shown significant improvement in mortality and life quality in levocardia patients, but is significantly more challenging to implant and unstandardized in dextrocardia patients.^5–8^

In lieu of the rare number of literature-reported scenarios of CRT deployment in DXT SIT, we elaborate on a unique approach to the management of conduction system disease and heart failure in this case of DXT with SIT.

## Summary figure

**Table:** 

Timeline	Events
Day 1	A 56-year-old female with a history of dextrocardia (DXC) with situs inversus totalis (SIT), sick sinus syndrome (SSS) s/p left-sided Boston Scientific dual-chamber pacemaker placement, paroxysmal atrial fibrillation (pAF) s/p pulmonary vein ablation (PVI), and tachycardia/pacemaker-induced heart failure with improved ejection fraction (HFimEF) 44% presents for shortness of breath and chest discomfort during exertion. On physical exam, noted to be bradycardic.
Day 2	The electrocardiogram (ECG) shows dual atrial and ventricular pacing with prolonged atrioventricular (AV) conduction and a ventricular rate of 55 beats per minute. QRS duration was 202 ms. Echocardiogram shows moderate left ventricular (LV) dilation with EF 44%. Pacemaker interrogation reveals the right ventricular (RV) lead is functional, although pacing thresholds are elevated and bipolar impedance is chronically elevated.
Day 5	Cardiac resynchronisation therapy pacemaker (CRT-P) placement is performed under moderate sedation. A dedicated right-sided Medtronic ATTAIN Select II™ sheath is utilized for CS cannulation guided by a multipolar electrophysiology catheter. A bipolar Medtronic CS lead is used to ensure compatibility with the chronic RV lead. Boston Scientific Valitude CRT-P model U125 is connected to the proximal portions of the pacing leads.
Day 6	Postoperative ECG demonstrates a QRS duration of 142 milliseconds. Posteroanterior (PA) chest x-ray demonstrates an acceptable CS lead position after posterolateral vein cannulation.
Day 365	Device Interrogation Reveals 99% Biventricular Pacing and an LV Lead with Good Pacing Threshold and Impedance. RV Lead Is Still Functional With The LV Lead In Backup. Repeat Echocardiogram Shows EF 49%. Patient Reports Functional Improvement And Ability To Tolerate Moderate Level Activities.

## Case presentation

The patient was a 56-year-old female with past medical history of dextrocardia (DXC) with situs inversus totalis (SIT), sick sinus syndrome (SSS) s/p left-sided Boston Scientific dual-chamber pacemaker placement, hypertension, paroxysmal atrial fibrillation (pAF) s/p pulmonary vein ablation (PVI), and nonischemic cardiomyopathy heart failure with improved ejection fraction (HFimpEF) 44%.

The patient presented to the electrophysiology clinic for evaluation of shortness of breath and chest discomfort that occurred predominantly on moderate exertion, consistent with New York Heart Association (NYHA) class II symptoms. 1 year prior to presentation, she had presented with episodic palpitations and shortness of breath at rest, as well as a decrease in exercise tolerance, which were diagnosed as pAF and heart failure with reduced ejection fraction (HFrEF) 35%. Prior to this presentation, cardiac function and left ventricular ejection fraction (LVEF) were in the normal range of 50–55%. Apart from the comorbidities described above, no other chronic degenerative diseases of the heart were identified. Over time, her clinical course was complicated by progressive atrioventricular conduction abnormalities and recurrent atrial fibrillation with ventricular pacing burden ranging from 85% to 99%. Concurrent with the development of ventricular pacing dependence and sustained periods of atrial fibrillation, she developed symptoms of heart failure, and the LVEF fell to 35%. In lieu of the suspected tachycardia-induced cardiomyopathy, the patient subsequently underwent direct current cardioversion (DCCV). However, as DCCV did not abate symptoms or improve cardiac functionality in the long term, the patient then underwent PVI with initiation of dronedarone 400 mg twice daily and apixaban 5 mg twice daily, resulting in subsequent improvement of symptoms and LVEF. Guideline-directed medical therapies started at this time included metoprolol succinate 25 mg daily and furosemide 20 mg daily. 6 months before presentation, echocardiogram showed heart failure with improved ejection fraction (HFimpEF) 44% (see Supplementary material online, *Video S1*). 3 years before presentation, pro-brain natriuretic peptide was 158 pg/mL, and at presentation it was 264 pg/mL (normal <100 pg/mL).

## Physical exam

Vitals were temperature 97.8 F, heart rate 59 beats per minute, blood pressure 121/79 mmHg, respiratory rate 17 breaths per minute, and SpO2 98% on room air. On exam, the pacemaker device was intact in the left pectoral pocket with a well-healed incision site. Bradycardia was appreciated on heart sounds. Lung sounds were normal. The rest of the exam was normal.

## Imaging

**Figure 1:**
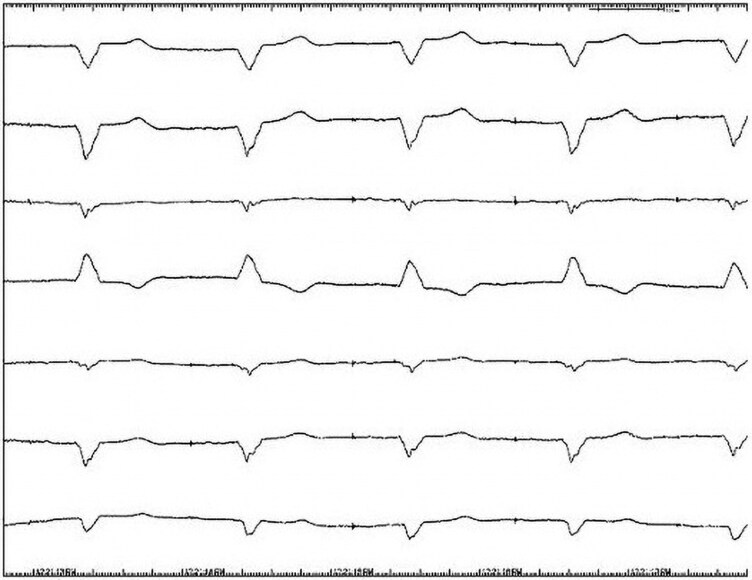
ECG tracings prior to CRT-P implantation showing QRS interval of 202 ms.

The electrocardiogram (ECG) showed dual atrial and ventricular pacing with prolonged atrioventricular (AV) conduction and a ventricular rate of 55 beats per minute. QRS duration was 202 ms (*Figure 1*). Echocardiogram showed moderate left ventricular (LV) dilation with an ejection fraction of 44%.

Pacemaker interrogation revealed that the right atrial (RA) Guidant lead pacing was at 100%, and right ventricular (RV) St. Jude lead pacing was at 99%. The RV lead was functional, although pacing thresholds were elevated, and the bipolar impedance was chronically elevated. There was dual chamber pacing at 70–120 bpm.

## Management

**Figure 2:**
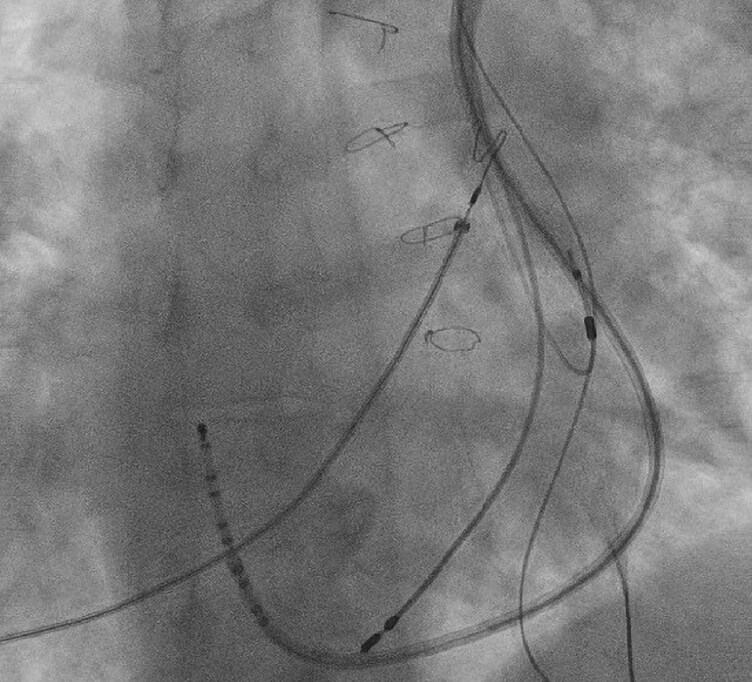
Right Medtronic ATTAIN select II catheter CSC with multipolar catheter localising CS.

**Figure 3:**
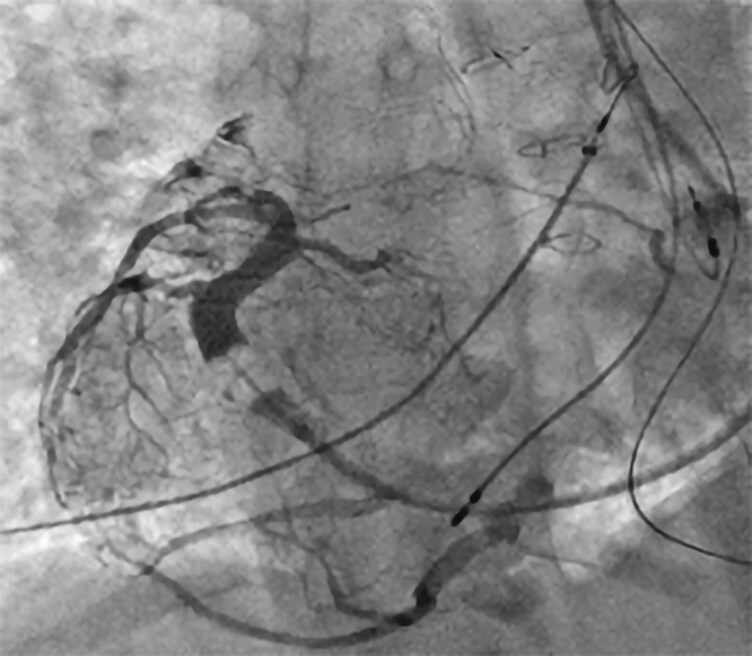
CS venogram after cannulation with multipolar catheter.

The procedure was performed in an attempt to upgrade the dual-chamber pacemaker system to a cardiac resynchronisation pacing system (CRT-P). Moderate sedation was given. Left upper extremity venography demonstrated patent left axillary and left subclavian veins. Venographic and ultrasound techniques were used to cannulate the subclavian vein via a micro-puncture technique. Identification of the coronary sinus and its ventricular branches was a challenge, given the patient's atypical anatomy. Under fluoroscopy, a guidewire was advanced into the central venous circulation and a dedicated right-sided Medtronic ATTAIN Select II™ sheath was utilized for CS cannulation guided by a multipolar electrophysiology catheter (*Figure 2*). A balloon-tipped catheter was then deployed through the introducer, and coronary sinus venography was performed (*Figure 3*). Numerous branches were identified, but most were associated with high pacing thresholds, phrenic nerve stimulation, or undesirable locations. Sub-selective catheters and specialized guidewires were utilized to finally allow for cannulation of an acceptable posterolateral vein location.

**Figure 4:**
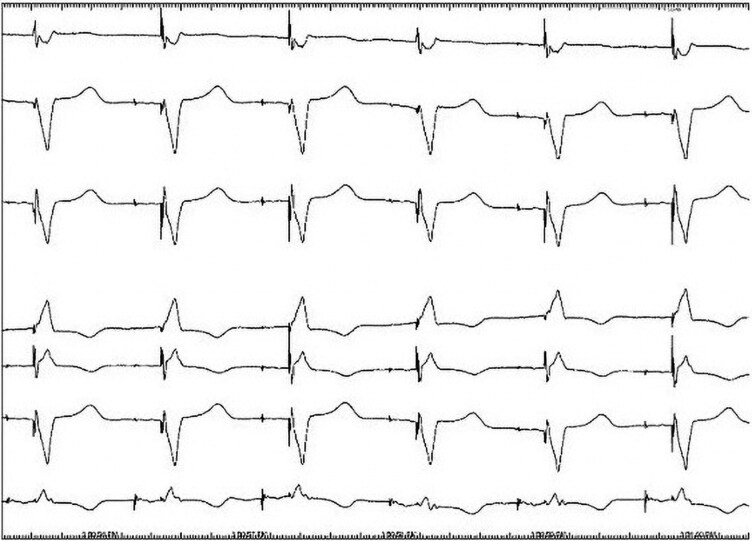
ECG tracing after CRT-P implantation showing QRS interval of 142 ms.

**Figure 5:**
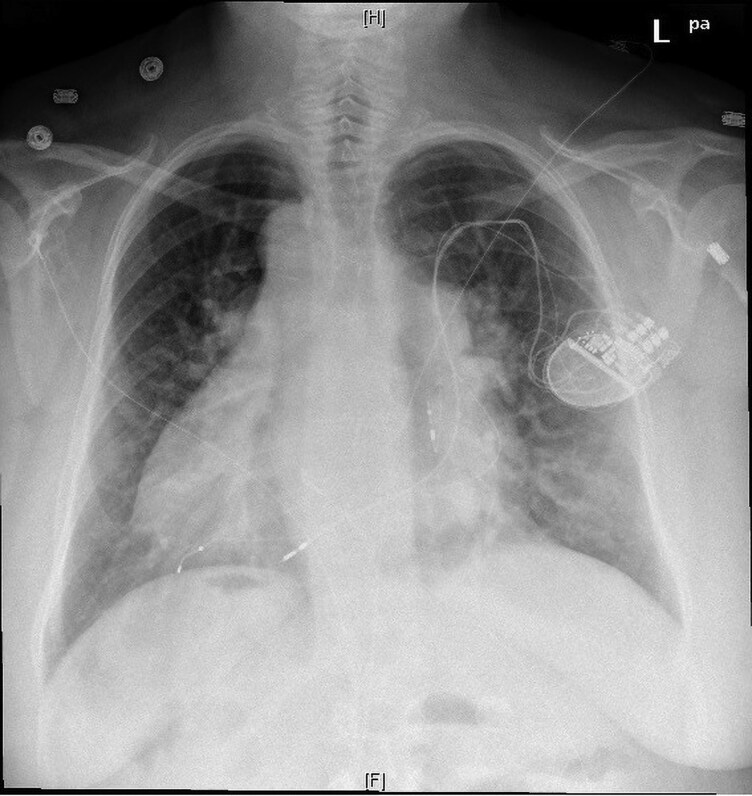
PA chest x-ray demonstrating CRT-P implant with bipolar LV lead and lead positions.

A bipolar, rather than quadrapolar, CS lead was utilized to ensure that it could be inserted into the IS-1 RV port, in the event that the chronic RV lead failed. Multi-vectored sensing and pacing thresholds were obtained, and the lead position was assessed for diaphragmatic stimulation. Once satisfactory thresholds were obtained, the lead was secured proximally. The final lead position assessed fluoroscopically and was felt to be at the mid-lateral aspect of the left ventricle. Boston Scientific Valitude CRT-P model U125 was then connected to the proximal portions of the pacing leads. The postoperative ECG demonstrated a QRS duration of 142 milliseconds (*Figure 4*). Posteroanterior (PA) chest x-ray demonstrated an acceptable CS lead position after posterolateral vein cannulation (*Figure 5*). Anticoagulation with apixaban was maintained during the procedure. It was only discontinued for 48 h after the procedure, following which it was resumed along with the remainder of the patient's home medications.

At 6 weeks after CRT-P placement, device interrogation showed impedances were 403 ohms, 1804 ohms, and 1093 ohms in the atria, right ventricle, and left ventricle, respectively. Thresholds were 0.7 V at 0.4 ms, 2.8 V at 0.4 ms, and 0.6 V at 0.4 ms at the atria, right ventricle, and left ventricle, respectively. At 1 year post procedure, device interrogation revealed 99% biventricular pacing and an LV lead with good pacing threshold and impedance. The RV lead was functional with the LV lead as backup in case the former failed. A repeat echocardiogram 2 months after the procedure showed LVEF 49% (see Supplementary material online, *Video S2*). The patient also reported functional improvement and the ability to tolerate moderate-level activities. Valsartan sacubitril 24–26 mg twice daily was eventually added to the heart failure regimen, given stable vital signs and lab markers. However, the patient’s subsequent home medication regimen resulted in low normal vital signs and, in particular, a low heart rate. The decision, therefore, was made to avoid further optimisation of heart failure treatment beyond the addition of valsartan sacubitril. Moreover, the thrombotic risk was calculated based on CHADSVASC with the patient’s score (including gender) at 3 (high risk for stroke). The hemorrhagic risk was calculated based on HASBLED, with the patient’s score at 1 (low risk for bleeding).

## Discussion

Historically, CRT-P has been performed in very few instances of literature-reported cases involving DXC and SIT. Previous approaches to CRT-P have utilized several anatomical sites and cannulation approaches. Among them have been the use of long electrophysiology sheaths in the CS, followed by the advancement of bipolar leads into the posterolateral branch of the CS.^3^ Others have included the cannulation of the innominate vein with a pair of telescoping sheaths and subsequent LV lead placement in a posterior lateral branch.^6^ Moreover, other approaches have used a variety of technical steps, including counterclockwise rotation of a deflectable D-type quadripolar electrophysiology catheter to reach the CS ostium and entering a left lateral marginal vein to deliver a quadripolar LV lead.^4^ Still others have used more innovative strategies, including image reversal to mimic levocardia, clockwise maneuvering of the delivery system, and delivering the LV lead to a lateral branch vein.^1,8^ Thus far, only a few studies have utilized a bipolar LV lead in the CS.^9–14^

Our case is among the few in the literature demonstrating the successful but technically challenging approach to CRT-P implantation in DXC. The distinguishing factors in our case were the use of a right-sided delivery system through a left venous access point and subsequent use of a bipolar CS lead. Considering the reversal of anatomy, a right sided system delivery system was felt to be the preferred approach to reaching the CS in this patient, whose pacing system was implanted from the left, as the course to the CS was more analogous to a right-sided implant in a patient with normal cardiac anatomy. Also somewhat counterintuitive, we did not utilize a quadripolar CS lead, which typically provides multiple vectors to enhance pacing function and avoid phrenic nerve stimulation. The use of a bipolar CS lead in this specific case was to facilitate management of this patient in the event that her older, somewhat compromised RV lead failed in the future. This bipolar CS lead has a standard IS-1 connector and is compatible with any standard pacing port.

## Conclusion

CRT-P implantation in DXC is a challenging feat which can be accomplished more easily with the use of a right-sided CRT-P delivery system in the presence of a left-sided device. Given the arduous and technically difficult feat involved with CRT-P in DXC cases, operators must be prepared to utilize non-traditional methods and tools to achieve technical success in patients with this challenging anatomy.

## Lead author biography


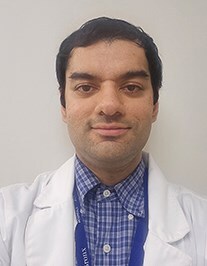


Dr Sujoy Khasnavis is an internal medicine graduate of Jacobi North Central Bronx Hospital.

## Supplementary Material

ytag167_Supplementary_Data

## Data Availability

All data are incorporated into the article and its online Supplementary material.

## Notes

**Consent:** In line with COPE guidelines, informed consent was obtained from all identifiable individuals, along with their right to withdraw consent. Participants understood the potential implications of their information being published, and they freely agreed to publication. Appropriate measures were taken to anonymize details where necessary.
